# Evaluation of request forms submitted to the haematology laboratory in a Ghanaian tertiary hospital

**DOI:** 10.4314/pamj.v8i1.71148

**Published:** 2011-03-29

**Authors:** Edeghonghon Olayemi, Rebecca Asiamah-Broni

**Affiliations:** 1 Department of Haematology, University of Ghana Medical School, University of Ghana, Ghana; 2 Department of Medical Laboratory Sciences, School of Allied Health Sciences, University of Ghana, Ghana

**Keywords:** Evaluation, request forms, haematology Unit, Ghana, Tertiary Hospital

## Abstract

**Background:**

Laboratory request forms provide information about the laboratory test being requested for. They carry demographic data and other information such as location of patient, laboratory number, doctor’s name, signature of the doctor, telephone number of the requesting doctor. Omission of information on the forms may lead to laboratory errors. The aim of this study was to evaluate the level of completion of laboratory request forms at the haematology department of a Ghanaian tertiary hospital.

**Methods:**

Three thousand request forms submitted to the haematology department between January and April 2010 were retrieved and studied. The information provided on each request form was recorded in a spread sheet and analyzed.

**Results:**

The patient’s age and sex were missing in 25.6% and 32.7% of the forms respectively. About half of the request forms did not have the patient’s location. No clinical detail was provided on 22.7% of the forms. Doctors were more likely to sign their request forms and provide a name but they all failed to provide an address or a contact telephone number. Conclusion: This study demonstrates that, the standard of completion of request forms was poor. Essential information required on the forms was often missing. This can lead to limited advice given by laboratory physicians and may increase the potential for errors. Conversely, provision of all the information needed on the forms will aid laboratory diagnosis and enhance patient care and save time and resources. There should be closer interaction between clinicians and laboratory personnel to improve quality of services.

## Background

Audit has been defined as a quality improvement process that seeks to improve patient care and outcomes through systematic review of care against explicit criteria and the implementation of change [1]. It is a part of continuous quality improvement process and a key element of clinical governance. Laboratory- based audits evaluate components of laboratory services; providing feedback to staff and users of the laboratory [2]. There are three components involved in laboratory auditing, these are: pre-analytical, analytical and post-analytical. Evaluation of laboratory request forms is a pre-analytical audit. Medical errors are known to impact negatively on patient’s outcome [3]) and modern medical practice is increasingly dependent on reliable clinical laboratory services [4]. It has actually been demonstrated that laboratory results influence up to 70% of medical diagnoses [5].

In a study in Italy out of a total of 393 questionable findings on results from the laboratory, 160 were confirmed as laboratory errors of which,

61.9% were pre-analytical, 15% were analytical and 23.1% were post-analytical errors [3].

Previously, laboratories focused their attention on eliminating or reducing errors in the analytical phase [4]). However, it has been demonstrated that currently, pre- and post-analytical processes in the laboratory are more vulnerable to errors than the analytical steps [6]. In a report by Plebani and Carraro, up to 68% of laboratory errors occurred in the pre-analytical phase [7]. This phase includes procedures which are not under the control of laboratory personnel and are mostly performed outside the laboratory; such as: completion of laboratory request forms, specimen identification, phlebotomy, sample handling and transportation to the laboratory [8].

Laboratory request forms provide information about the laboratory test requested for. They contain demographic data such as name(s), date of birth, subject’s address, age, and sex. Other details include the ward, laboratory number, doctor’s name, signature of the doctor, telephone or fax number of the doctor; clinical details, fasting status of the subject and the date of request. In the Hospital we studied, laboratory request forms are filled by doctors and presented to the laboratory.

From the results of a research carried out in South Africa, it was observed that laboratories were experiencing significant problems with incompletely filled request forms and the standard of completion of request forms was poor and represented a threat to patient safety and quality of laboratory services [9]. In Ghana, there has been no study on the trend of incompletely filled laboratory request forms. Audit of laboratory request forms presented to the laboratories will provide valuable information that will assist both laboratory physicians and clinicians in improving the standard and quality of laboratory results. This is expected to also impact positively on patient care.

The objectives of this study were to evaluate request forms submitted to the haematology laboratory to determine the frequency of incompletely filled forms and the information that was overlooked.

## Methods

The audit was carried out at the haematology department of a tertiary hospital in Ghana. It was a retrospective study conducted on all request forms submitted to the haematology laboratory between January and April, 2010. Ethical approval was obtained from the ethics committee of the school of allied health sciences, university of Ghana. Each request was assessed for completeness of information supplied.

### Data collection

Three thousand consecutive request forms submitted to the haematology department between January and April 2010 were retrieved and studied. The information provided on each request form was recorded in a spread sheet and evaluated using SPSS version 16.1 Patient’s confidentiality was maintained. No identifying information (name, hospital identification number) was included on the data record sheet and patients were identified by a study number only. A frequency distribution table was created to summarize the data. The type of data collected is shown in Table 1.

## Results

A total of three thousand request forms were studied. Table 1 shows the information required on laboratory request forms and the percentage of laboratory forms that contained the required information.

Out of all the required information only the patient’s name and the laboratory test being ordered were present on all 3,000 forms.

The patient’s age was not supplied on 769 (25.6%) forms, while patient’s gender was present on 67.3% of forms. The location of the patient was missing on 1433 (47.8%) forms. Only 77.3 % of the request forms evaluated contained the clinical details of the patient.

With respect to physician information; the name of the physician ordering the test was provided on 55.4% of forms, while 75.7% were signed by the doctor. The date the test was ordered was present on 62.7% of forms

None of the 3,000 request forms had the requesting physician’s telephone / fax number or the time the specimen was collected.

## Discussion

The healthcare system is increasingly dependent on reliable clinical laboratory services. However, few audits of the pre-analytic phase have been carried out [7]. In this study, we evaluated the level of completion of laboratory request forms.

Our evaluation revealed that, only the clients name and the investigation requested appeared on all the forms evaluated, this is comparable to a similar study by Burton and Stephenson [10], where only the patient’s full name was stated on all the 2000 request forms they evaluated. This was not surprising since it was very likely that the request would have been turned down if the required test was not stated and the client’s name was absent.

Other demographic details such as the age and gender were not stated on 25.6% and 32.7% request forms respectively this is higher than figures of 5.8% and 14% respectively obtained from a study in Pakistan [11]. The patient’s location was missing in 47.8% of the request forms as compared to 4.9% obtained by Nutt et al in South Africa in 2008 [5]. The patient’s demographic data is relevant because it helps in specimen identification and proper interpretation of results.

In instances where samples from different subjects have the same or similar names, information such as the location of the subject, age and gender are important in identifying and sorting out both the subject and samples. Also reference ranges for some tests like the haemoglobin concentration vary with age and gender. The location/ward of the patient enables urgent results to be immediately communicated to the clinician.

No clinical detail was provided on 22.7% of the request forms sampled. This was comparable to results obtained in a similar study conducted by Nutt et al [5] in South Africa but higher than results obtained in the United states in a study by Nakleh et al [12]. In these studies, 19.1% and 2.4% of the request forms examined did not have the clinical information. The result obtained from this study was however lower than that from Pakistan where 34% of the request forms had no clinical information supplied [11]. Burton and Stephenson have demonstrated that, provision of adequate clinical information prevents inappropriate investigations. Absence of clinical information or misleading information leads to extraneous and unnecessary additional tests which has definite resource management and demand implications [10]. Also where interpretative comments are made on laboratory results, inadequate clinical information may lead to misleading and potentially harmful comments [13].

Most clinicians (75.7%) signed the request forms presented to the laboratory. However, only 55.4% indicated their names. The clinicians’ contact telephone numbers were the most incomplete parameters provided. In this study, none of the clinicians provided their contact information compared to almost 40% in the South African study [5]. Urgent results can be rapidly conveyed back to the requesting clinician if the contact telephone number is present on the request forms [11].

The date on which the requests were made was found on 62.7% of all the request forms. None of the request forms carried the time the sample was taken, this may be because the usual practice at the study site was to enter the time on the specimen bottle itself.

## Conclusion

This study demonstrates that, the standard of completion of request forms at our study site is poor. The request forms did not contain adequate demographic data of the subjects. Clinical details of the patients were not supplied on an appreciable number of the request forms. Details of the requesting clinicians were also lacking. The date on which the request was made was absent on most request forms. We recommend that there should be closer interaction between laboratory personnel and clinicians. Medical students should be adequately exposed to the medical laboratory and how it functions; this will help them understand the complimentary roles played by clinical and laboratory practice. The laboratories should be more closely involved in organizing orientation programs for newly employed doctors, especially pre-registration house officers. At such programs the importance of providing all relevant information to the laboratories for the right diagnosis to be made would be re-emphasized. This will benefit the patient in his/her care and management; the doctor, the laboratory and the hospital as well. As a last resort patient’s samples accompanied by incompletely filled request forms should be rejected since it may lead to inappropriate diagnosis. We recommend a repeat evaluation after one year, to assess if there has been any improvement in the standard of completion of request forms. A limitation of this study was our inability to assess the effect if any that this poor rate of completion had on patient’s management. This is a possible area for future research.

## Competing interests

The authors declare no competing interests

## Authors’ contributions

Edeghonghon Olayemi conceived and designed the study, was involved in the literature review, writing and editing the final draft of the paper. Rebecca Asiamah-Broni collected the data, carried out the data and statistical analysis and was involved in literature review, writing and editing of the final draft. Both authors approved the final version of the manuscript.

## Figures and Tables

**Table 1:** Information required on laboratory request forms and their completion rates in an evaluation of request forms submitted to the haematology laboratory in a Ghanaian tertiary hospital

Information Required	Percentage Completion Rate
Patient’s Name	100.0
Laboratory Request	100.0
Clinical details	77.3
Doctors Signature	75.7
Age	74.4
Gender	67.3
Date of Request	62.7
Doctor’s Name	55.4
Patient’s Location	52.2
Telephone/ Fax Number	0.0
Time of Request	0.0
